# Proof-of-Concept Vacuum Microelectronic NOR Gate Fabricated Using Microelectromechanical Systems and Carbon Nanotube Field Emitters

**DOI:** 10.3390/mi14050973

**Published:** 2023-04-29

**Authors:** Tasso von Windheim, Kristin H. Gilchrist, Charles B. Parker, Stephen Hall, James B. Carlson, David Stokes, Nicholas G. Baldasaro, Charles T. Hess, Leif Scheick, Bernard Rax, Brian Stoner, Jeffrey T. Glass, Jason J. Amsden

**Affiliations:** 1 Department of Electrical and Computer Engineering, Duke University, Durham, NC 27708, USA; 2 RTI International, Research Triangle Park, NC 27709, USA; 3 Micross Advanced Interconnect Technology, Research Triangle Park, NC 27709, USA; 4 Department of Physics, University of Maine, Orono, ME 04469, USA; 5 Jet Propulsion Laboratory, California Institute of Technology, La Canada Flintridge, CA 91011, USA

**Keywords:** carbon nanotubes, field emission, logic gate, MEMS, vacuum microelectronics

## Abstract

This paper demonstrates a fully integrated vacuum microelectronic NOR logic gate fabricated using microfabricated polysilicon panels oriented perpendicular to the device substrate with integrated carbon nanotube (CNT) field emission cathodes. The vacuum microelectronic NOR logic gate consists of two parallel vacuum tetrodes fabricated using the polysilicon Multi-User MEMS Processes (polyMUMPs). Each tetrode of the vacuum microelectronic NOR gate demonstrated transistor-like performance but with a low transconductance of 7.6 × 10^−9^ S as current saturation was not achieved due to a coupling effect between the anode voltage and cathode current. With both tetrodes working in parallel, the NOR logic capabilities were demonstrated. However, the device exhibited asymmetric performance due to differences in the CNT emitter performance in each tetrode. Because vacuum microelectronic devices are attractive for use in high radiation environments, to test the radiation survivability of this device platform, we demonstrated the function of a simplified diode device structure during exposure to gamma radiation at a rate of 45.6 rad(Si)/second. These devices represent a proof-of-concept for a platform that can be used to build intricate vacuum microelectronic logic devices for use in high-radiation environments.

## 1. Introduction

Since the invention of the transistor in 1949, solid-state technology has dominated the electronics industry [1]. Due to its low cost and scalable integration, solid-state electronics quickly replaced vacuum tube-based technologies for most applications. Despite the performance benefits of solid-state technology, they are not well suited for use in high radiation environments [2]. Ionizing radiation can introduce charge states at transistor oxide boundaries, shifting the transistor threshold [3]. Vacuum microelectronic devices (VMDs), however, do not suffer from these issues because performance is not dependent on charge states. Like vacuum tube amplifiers (hereafter, simply vacuum tubes), VMD functionality relies on electron transport through a vacuum from the cathode to the anode. However, VMD and vacuum tube amplifiers use different sources for electron emission [4].

Vacuum tubes were widely used in the communication industry and early computers, but with the invention of the solid-state transistor [5] and integrated circuits in the 1940s and 1950s [5,6], vacuum tubes were largely replaced in computing applications. Over the next three decades, advances in microfabrication processing technology and field emission technology gave rise to vacuum microelectronic devices where the vacuum devices are micron-scaled [7,8,9]. Vacuum microelectronic devices are promising in areas where solid-state devices have limitations, particularly in harsh environments, i.e., high temperatures and intense radiation. Further, there are applications where vacuum microelectronics offer the possibility of unique capabilities or best-in-class performance, including Hall effect thrusters, electrodynamic tethers, traveling wave tubes, space satellite communication and various types of radar, and finally, key elements for the VMD-based integrated circuits [4,10,11,12,13].

Vacuum tube amplifiers typically employ metallic filaments that are heated sufficiently to induce thermionic emission of electrons, while VMDs utilize cold cathodes that emit electrons under the influence of an electric field, the phenomenon known as field emission [14,15]. This distinction is important as cold electron emission allows for miniaturization and reduces power consumption in VMDs compared to vacuum tubes. The power required to heat the cathode makes vacuum tube devices less efficient, and heat dissipation requirements limit the ability to miniaturize these devices [16]. VMDs, however, have no heating requirements, so they can be scaled down several orders of magnitude smaller than even the smallest vacuum tubes. Furthermore, modern VMDs utilize cathodes with high aspect ratio emitters, such as nanocrystalline diamond [17], carbon nanotubes [18], and metallic Spindt emitters [19] to allow for field emission at relatively low applied electric fields, reducing power consumption.

Carbon nanotubes (CNTs) are good candidates for field emission cathodes due to their geometry and robust material properties [15,20,21]. The high aspect ratio of CNTs enhances field emission, and the robust nature of CNTs can enable current densities of greater than 13 A/cm^2^ [22]. In addition to their favorable field emission performance, CNTs are good candidates for VMD cathodes because of their resistance to degradation in extreme environments, such as those with ionizing radiation. For example, Francis et al. determined using Raman spectroscopy that electron irradiation doses up to 10^17^ e/cm^2^ had no effect on the intrinsic structure of carbon nanotubes [23]. Research by Gupta et al. on micro- and nanocrystalline carbon, which share the same sp^2^ hybridized carbon with CNTs, shows a decrease in field emission turn-on field for increasing radiation levels, and their data suggest that nanocrystalline carbon tends to reach a state of damage saturation at Mrad radiation levels. [24]. These properties make CNTs a promising material for field emission cathodes in VMDs for use in radiative environments. However, the field emission performance of CNTs has not been studied while being exposed to radiation.

To provide a viable alternative to solid-state devices in extreme environments, a scalable manufacturing process capable of producing a wide variety of circuit elements on a single substrate is needed. Polysilicon surface micromachining is a well-established technique for fabricating a wide range of geometrically complex microelectromechanical systems (MEMS) structures, including freestanding structures extending outward from the substrate [25]. Fabricating devices from freestanding polysilicon panels provides many benefits for an integrated vacuum circuit platform. This fabrication technology is highly versatile because the number of process steps does not increase with increasing device complexity or increasing numbers of devices. All device structures are formed on a single substrate, so there is no need to align external components or integrate multiple substrates. Freestanding panel structures also offer the advantage of low capacitance to ground and, therefore, high-speed capability. Supporting elements such as resistors and inductors can be easily incorporated into this technology and can also be made freestanding. Our group has established a method to integrate carbon nanotube field emission cathodes into this platform [26,27,28]. 

Thus far, we have demonstrated a variety of devices, including triodes [28,29], ion sources [30,31] and bipolar microelectronic devices [32], and demonstrated that several devices could operate independently on the same substrate [33]. The triode devices consisting of a cathode with integrated CNT emitters, an extraction grid, and an anode exhibit transistor-like performance and are capable of a DC amplification factor of up to 600 and a transconductance of 2 µS. The extraction grid is biased positively relative to the cathode, which induces electron emission toward the anode. However, field emission is susceptible to large current fluctuations due to the effects of adsorbates that can change the local work function of the CNTs [34]. For this reason, to extract a constant current from the cathode, the extraction grid voltage must be varied constantly to compensate for changes in field emission current. This is disadvantageous in a triode device because the current reaching the anode cannot be controlled by a single voltage input. 

In this paper, we describe the fabrication and testing of a vacuum microelectronic NOR logic gate incorporating two parallel vacuum microelectronic tetrodes. These tetrodes are similar to the previously demonstrated vacuum microelectronic triodes but include an additional grid between the extraction grid and anode, called the control grid. The vacuum microelectronic tetrode design is similar in structure to the screen-grid tube, one of the first tetrodes invented in the early 1900s; however, the additional grid in our device differs in function. In the screen-grid tube, the grid closest to the anode is used to shield capacitance between the input and output circuits [35]. In our tetrode, the control grid is used to control the current that reaches the anode from the extraction grid. Therefore, in our tetrode device, the extraction grid voltage can be varied to generate a constant electron emission current from the cathode, and the control grid is biased independently to control the amount of current that reaches the anode, eliminating the issue with the triode device described above. This vacuum microelectronic NOR gate device represents the first vacuum microelectronic logic device built using the scalable MEMS platform with integrated CNT field emitters. We chose to demonstrate a NOR gate because it can be used in combination to replicate the functions of all other logic gates [36], making this device a crucial building block for vacuum microelectronic circuits. The transistor characteristics of the tetrode were analyzed, and the ability to perform logic was demonstrated. In addition, to test the radiation survivability of this device platform, we characterized the performance of a simple two-panel device, including a CNT field emission cathode on one panel and an anode for the second panel during gamma radiation exposure. We determined the effect of gamma radiation on CNT field emission performance using an in situ measurement approach not previously demonstrated. This approach provides valuable insight into how a device like a vacuum microelectronic NOR logic gate described in this paper would perform in a radiative environment.

## 2. Materials and Methods

### 2.1. NOR Logic Gate Fabrication and Testing

**Figure 1:**
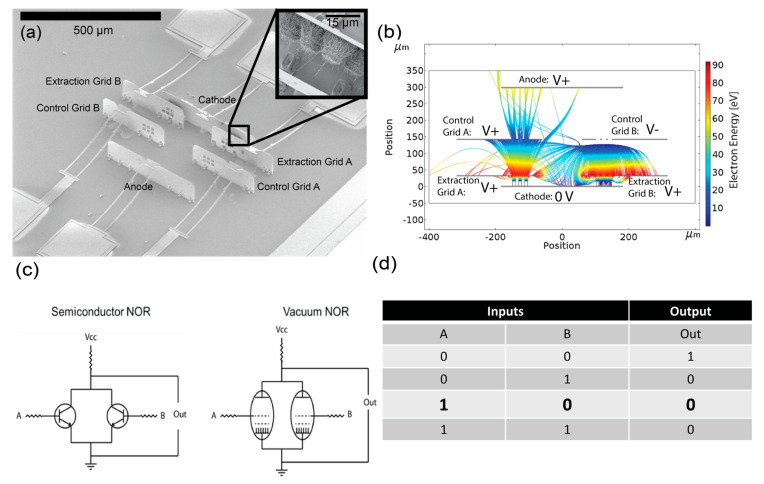
(**a**) SEM image of a MEMS NOR gate with CNT cathode shown inset. (**b**) Example electron trajectories from COMSOL simulations for a device with one control grid biased to allow electron flow through the control grid and one control grid biased to block electron flow. (**c**) Schematic of the vacuum NOR gate and semiconductor NOR gate. (**d**) An input/output diagram for a NOR gate with the conditions from the simulation is shown in (**c**) in bold text.

We fabricated the microelectronic NOR shown in Figure 1a. Prior to fabrication, we simulated electron trajectories to validate operational feasibility. Figure 1b shows a top-down schematic of the NOR gate with simulated electron trajectories. Electron trajectories were drawn using a 2D COMSOL simulation on a plane midway between the substrate and the top of the MEMS panels using the AC/DC and charged particle tracing modules. To approximate electron emission from the CNTs, a uniform surface emission model was chosen for the simulations in which the electrons were uniformly emitted from the top surface of the CNT emitter array bundles.

The vacuum microelectronic NOR gate consists of two parallel vacuum tetrodes with a common cathode and a common anode. The common cathode has two CNT field emitter arrays. The inset in Figure 1a shows a magnified view of one of the CNT arrays grown on the cathode. Electron emission from each CNT array is independently controlled by applying a positive bias to either of the two extraction grids. In addition, two control grids are independently positively or negatively biased to either allow or block electrons from reaching the anode. When the potential on the control grid is positive, electrons pass through the grid, and the circuit between the cathode and anode is closed. When the potential on the control grid is negative, electrons are blocked from reaching the anode, leaving an open circuit. In this way, our device operates similarly to a semiconducting NOR device, where each control grid is analogous to the gate of a transistor, controlling the conduction of electrons between the anode and cathode. Figure 1c shows a comparison of the vacuum microelectronic NOR gate and a semiconductor NOR gate. Figure 1d shows the truth table for a NOR gate with the conditions from the simulation in Figure 1b highlighted.

This device was fabricated using a well-established polysilicon MEMS process, polyMUMPs. Details of the fabrication process have been described in previous publications [18,25]. In brief, devices were fabricated by etching and micromachining a three-layer polysilicon substrate. Following the MEMS processing, to integrate CNT field emitters, a 5 nm thick iron catalyst layer was evaporated through a photomask onto the cathode in a 3 × 2 array pattern of squares 9 µm on a side. CNTs were grown on the cathode using a microwave plasma chemical vapor deposition, forming a 3 × 2 array of square CNT bundles. CNTs were grown at 850 °C using a 120-s ammonia catalyst pretreatment step and a growth step with a 3:1 ratio of methane to ammonia for 180 s. Pressure throughout the growth process is 21 Torr. After CNT growth, the panels were manually lifted perpendicular to the substrate and held in place with integrated latches. Each chip was then mounted and wire-bonded to a pin-grid array. 

The devices were characterized in vacuum at a pressure of ~5 × 10^−7^ Torr using Keithley 2410 source meters to apply voltage and measure the current at each panel of the device. Custom LabView software was used to record the current and voltage at each panel during device testing. To characterize the field emission performance of the CNTs field emitters, we swept the voltage of the extraction grid from 0-150 V relative to the cathode. The distance between the cathode and extraction grid is 33 µm, so this corresponds to an applied field of 0–4.5 V/µm. To account for variation in the cathode current, 10 voltage sweeps were conducted, and the results averaged together.

The performance of an individual tetrode was characterized prior to testing the full NOR gate. In the tetrode configuration, we examined the transistor-like performance. Electrons were extracted from the cathode by positively biasing the extraction grid relative to the cathode. Due to the noisy nature of CNT field emission, we used a hardware feedback loop in the Keithley 2410s that modifies the potential of each of the extraction grids to maintain a constant emission current from each side of the device. With a constant current maintained at the extraction grid, transistor curve data was obtained by setting a voltage input at the control grid and sweeping the anode voltage. The current at the anode was measured and repeated with incrementing voltages applied at the control grid. For NOR logic experiments, a constant current was maintained at the extraction grid, and a constant voltage was applied to the anode. The control grids were switched between positive to negative bias, and the current was measured at the anode to measure the ability of the control grid voltage to switch the anode current on and off on each side of the device.

### 2.2. In Situ Gamma Radiation Experiments

**Figure 2:**
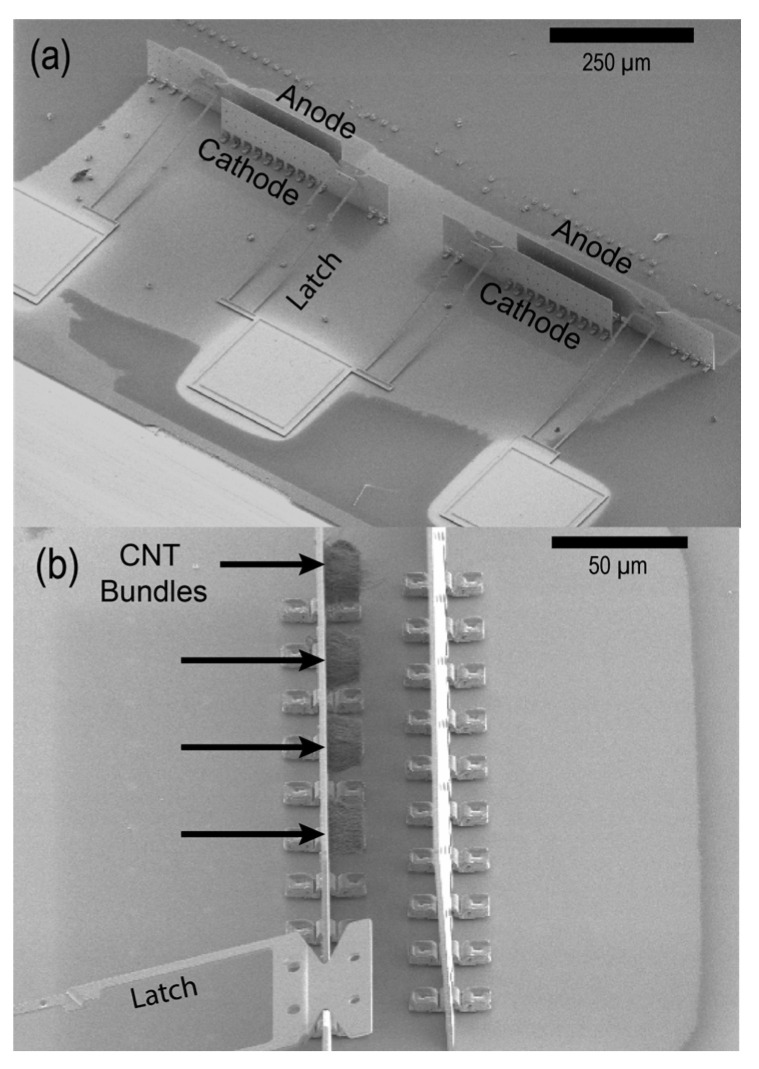
(**a**) SEM images of two two-panel devices taken at a 45° angle and (**b**) a further magnified image of a single anode and cathode with CNTs.

To test the radiation survivability of this device platform, we evaluated a simple two-panel device during exposure to gamma radiation at a rate of 45.6 rad(Si)/second for 900 s, for a total dose of 45,000 Rad. Each device consisted of two panels with a CNT-emitter cathode and planar anode separated by a gap of 35 µm. This simplified structure provided a convenient tool for evaluating the effects of radiation on CNT field emission and the physical structure. SEM images of a two-panel device are shown in Figure 2. Device fabrication and CNT growth were accomplished using the methods described above for the NOR gate.

Devices were tested in a vacuum chamber at 1 × 10^−3^ Torr placed in front of the cobalt-60 source. The pressure was limited by the need to place the vacuum pumps behind a radiation shield which required a long low conductance tube between the vacuum pump and vacuum chamber. The 45.6 rad(Si)/second source was cycled on and off with a period of approximately 5 min while the potential difference between the cathode and anode was varied to maintain a constant electron emission current of either 1 × 10^−8^ A and 5 × 10^−8^ A. Performance with and without radiation was evaluated by comparing the required variation in potential difference to maintain a constant electron emission current. Two devices were tested in this manner.

## 3. Results

**Figure 3:**
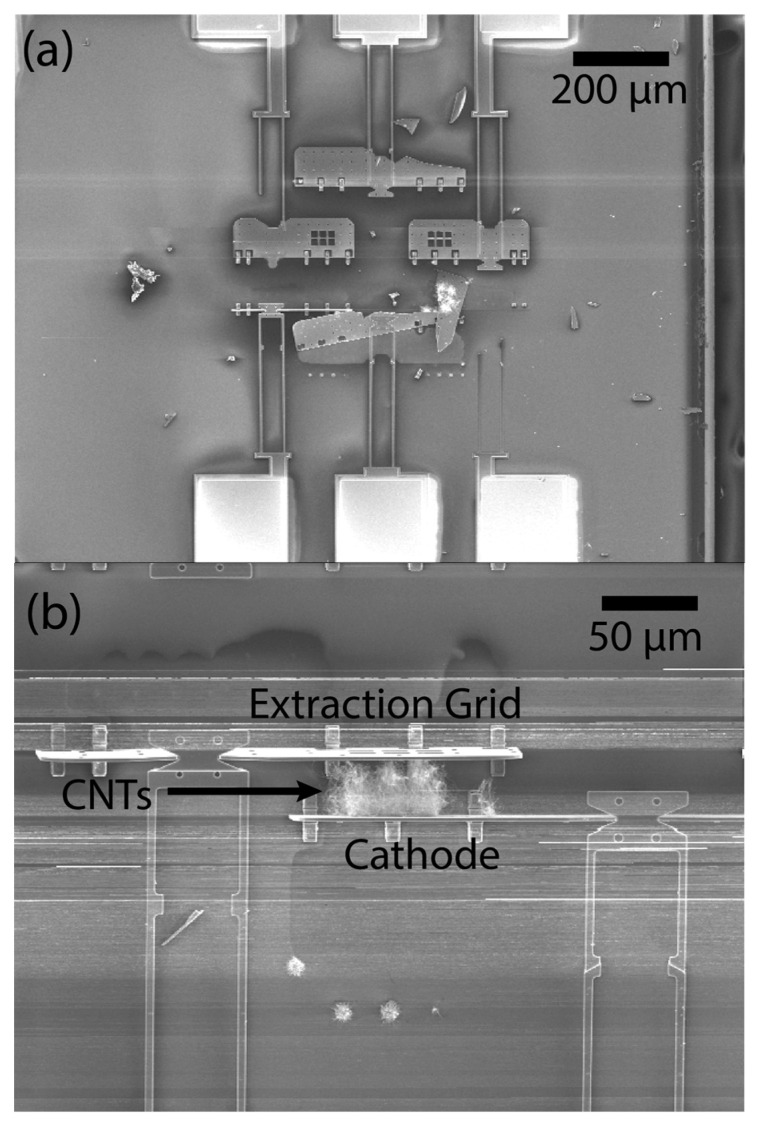
Common device failure modes. (**a**) The panels are broken during assembly, and (**b**) CNTs bridging the gap between the cathode and extraction grid.

Fabrication of the NOR device is not a trivial task, and several challenges were revealed during assembly, which resulted in low functional device yield. The causes were primarily open circuit failure due to broken panels and/or latches during assembly, as seen in Figure 3a, and short circuit failure due to CNTs bridging the gap between the cathode and extraction grid, shown in Figure 3b. Of these two failure modes, short circuit failure was more common and was due to inconsistent CNT growth between devices. Although the CNT growth process was the same for all devices, the resulting CNT morphology varied from device to device, leading to some devices having CNT bundles that were too long. Despite these challenges, we were able to fabricate a functional NOR device as well as a functional tetrode device.

### 3.1. CNT Emitter Characterization

**Figure 4:**
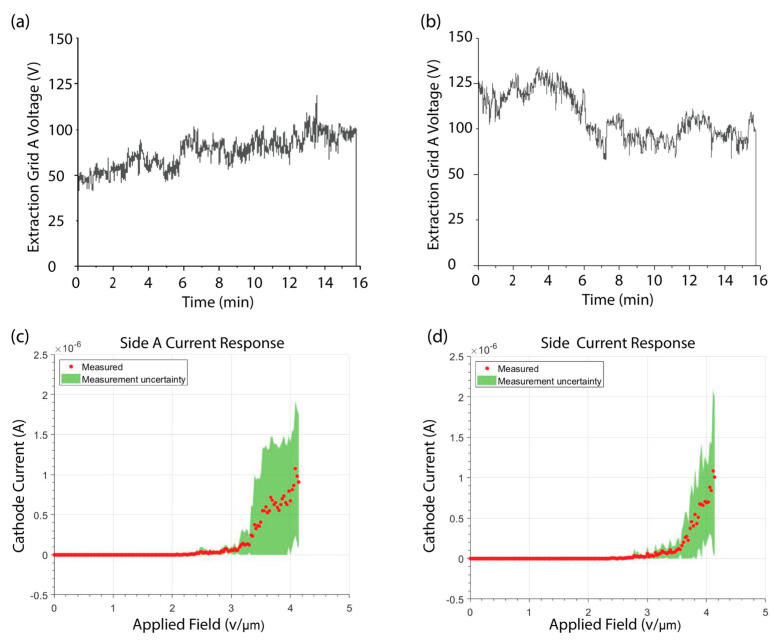
Voltage response needed to drive a constant current of 1 × 10^−7^ A for side A (**a**) and side (**b**) of the NOR gate. The cathode-to-extraction grid I-V characteristics for Side A (**c**) and Side B (**d**) of the NOR gate.

Figure 4 shows the performance of the CNT emitters on each side of the single fully functional NOR gate. Figure 4a,b show the extraction grid potential over time required to supply a constant current of 1 × 10^−7^ A. A stable current of 1 × 10^−7^ A was maintained with extraction grid A varying between 40 and 93 V and extraction grid B varying between 58 and 110 V. The wide variability in the voltage needed to drive a constant current is evidence of the inconsistent nature of field emission from an array of CNTs. This inconsistency is believed to be caused by local work function changes on CNT surfaces due to adsorbates from the environment adsorbing and desorbing on the CNT surface [34]. Reports in the literature have suggested mitigating this with a “burn-in” period, where a constant voltage is applied for several hours [37,38]. However, we observed no improvement in current stability, even after 24 h of operation.

Figure 4c,d show the IV characteristics for each side of the device. The measurement uncertainty, shown in green, represents the standard deviation of the cathode current for an average of 10 voltage sweeps. For side A, the turn-on field, the applied field necessary to induce electron emission, was 2.0 V/µm, while for side B the turn-on field was 2.2 V/µm. The difference in turn-on field and the large standard deviation of the 10 scans highlights the variability in field emission performance discussed above. The difference in performance between side A and side B is likely due to differences in the morphology of the CNT bundles at the respective sides of the cathode. Even slight differences in CNT morphology can affect field emission performance [37,38]. It is unclear what causes this difference in morphology, but differences in catalyst film thickness have been shown by Hofmann et al. to impact CNT bundle morphology [39]. A 5 nm-thick catalyst is evaporated through a shadow mask on each side of the cathode to control where the CNT bundles will be grown [27]. A slight misalignment of the shadow mask, or misalignment of the cathode itself, can lead to a difference in catalyst film thickness.

### 3.2. Tetrode Operation and Transistor Performance

**Figure 5:**
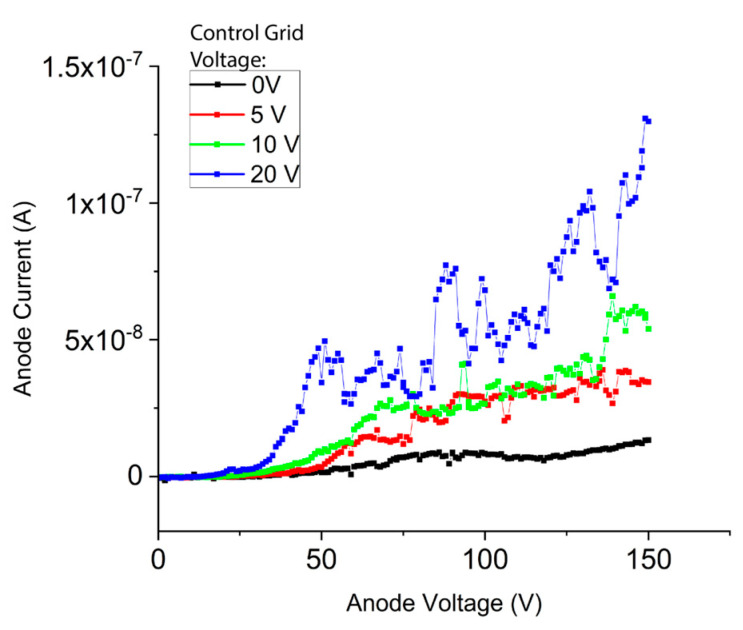
Plot of a representative transistor curve from one side of a NOR gate.

Figure 5 shows the transistor-like performance of a device with a single functioning tetrode. The extraction grid was varied to extract a constant current of 1 × 10^−6^ A from the cathode. The anode voltage was swept from 0–150 V with incrementing control grid potentials. The device demonstrated a trend of increasing anode current with increasing control grid potential. Transconductance, the measured change in anode current divided by the change in control grid voltage, of this device was 7.6 × 10^−9^ S when the cathode was grounded, anode potential was 150 V, and control grid voltage was incremented from 0 to 20 V. No change was observed for control grid voltages above 20 V for this device. Two tetrode devices were tested, and both performed similarly. Transistor performance was not measured for the NOR gate due to mechanical failure during testing.

The measured transconductance of these tetrodes is low compared to our previously reported triode device, which had a transconductance of 2 µS. This difference in transconductance is because only a small portion of current leaving the cathode reached the anode in the tetrode [28]. We measured an average of 7.5% of the cathode current collected at the anode, which significantly reduced the transconductance of the device. 

We were also unable to observe anode current saturation in these devices and therefore were unable to calculate the amplification factor of the device. The control grid voltage also has an unintentional effect on the cathode current. As anode voltage increases, the cathode current also increases, leading to an increase in anode current instead of current saturation.

### 3.3. NOR Gate Operation

**Figure 6:**
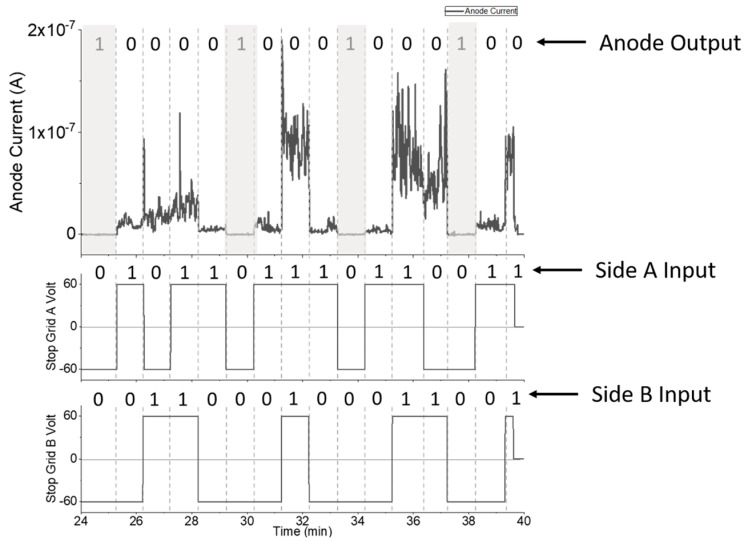
Demonstrating NOR gate operation with both tetrodes working in parallel.

Figure 6 shows NOR gate performance when voltages of ±60 V were applied to control grids A and B to modulate current at the anode, which was held at 60 V. The voltages for the control grid were determined based on the ability to stop all current from reaching the anode. A voltage of −60 V was required to stop electron flow to the anode, so operation at ±60 V was chosen to keep the on/off voltage conditions equal in magnitude.

When side A or B were “on,” the voltage applied to the control grid was 60 V, and there was current flowing at the anode. It was only when both A and B were “off” that there was no current at the anode. It should be noted that the current from sides A and B were not symmetric for reasons described in Section 3.1. The average anode current from side A of the device was approximately 6.8 × 10^−9^ A, while the average current from side B was 3.5 × 10^−8^ A. The average current when the device is “off” was 3.5 × 10^−10^, which translates to an on/off ratio of approximately 20 for side A and 100 for side B. Despite the differences in emission current and on/off ratio, the device functions as a NOR gate.

### 3.4. In Situ Gamma Irradiation Experiments

**Figure 7:**
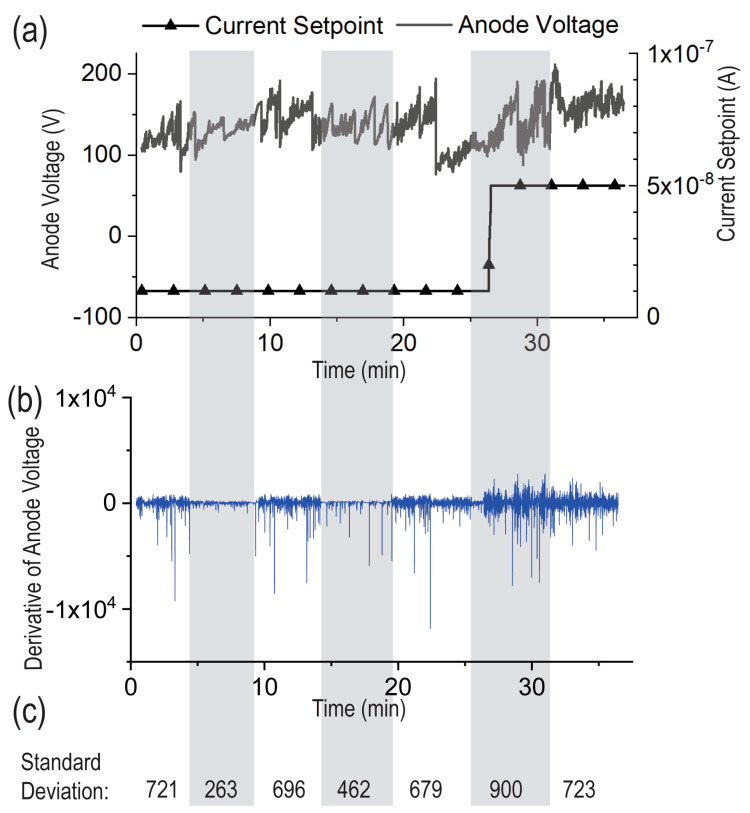
Anode voltage (**a**), standard deviation of anode voltage (**b**), for a two-panel device while radiation is switched on (grey sections) and off (white sections). (**c**) shows the standard deviation of the derivative of anode voltage for each section.

Figure 7 shows a plot of anode voltage for a representative two-panel MEMS device with a CNT cathode (see Figure 2) vs. time. The anode voltage was varied to maintain a current set-point of either 1 × 10^−8^ A or 5 × 10^−8^ A. As discussed above, the voltage variation required to maintain a constant emission current is a result of localized work-function changes due to molecules adsorbing and desorbing from the CNT surfaces, changes in CNT morphology, and physical damage to the field emitters [40]. The sample was exposed to gamma radiation at approximately 5 min intervals, as shown by the grey shading on the graph. 

For an emission current of 1 × 10^−8^ A, while the overall magnitude of voltage variation was similar when the radiation was on vs. off, the rate of variation in voltage was significantly less when the gamma radiation was on. We plotted the standard deviation of the derivative of the anode voltage vs. time to highlight the lower rate of variation under radiation exposure. However, for an emission current of 5 × 10^−8^ A, the rate of variation in voltage was roughly equivalent to the rate of variation when the radiation was off. A second device was also tested, which exhibited similar results (data not shown).

A control experiment taken with similar conditions, but in the absence of field emission, indicated a 7.7 × 10^−9^ A background current due to the ionization of residual gas in the vacuum chamber by the gamma radiation. As this background current is similar in magnitude to the current set-point, it is likely the cause of the reduced voltage variation required to maintain a constant emission current of 1 × 10^−8^ A. At the higher emission current of 5 × 10^−8^ A, the CNT field emission dominates the current at the anode, and the voltage variation required is similar with and without radiation.

## 4. Discussion

In this paper, we demonstrated two key capabilities of a vacuum microelectronic NOR device fabricated with the polyMUMPs process containing an integrated CNT field emission cathode and demonstrated the operation of a simple two-panel device while under exposure to gamma radiation. For the vacuum microelectronic NOR gate, the capability of current control and transistor-like functionality, as well as its ability to perform logic operations, were demonstrated. This device not only shows the increased complexity this platform is capable of, but it demonstrates proof-of-concept of one of the most important components of an integrated vacuum circuit. NOR gates can be used in combination to replicate the functions of all other logic gates, so this work provides a starting point for developing much more complicated circuits. Three main areas of performance need significant improvement before these devices can be widely used, including current loss at the extraction and control grids, improving the uniformity of CNT emission, and increasing robustness.

One promising method of improving electron transmission has been suggested in work done by Radauscher et al. [33]. In this paper, they suggest adding a collimation grid panel close to the cathode to reduce the angular dispersion of the emitted electrons. This method led to more than a 2× increase in anode current as grid loss was decreased from 58% to 23%. As these devices are built on the same platform as the device described in this paper, we can expect similar results by implementing a collimating grid into our design.

The asymmetric performance of the device will also need to be addressed in future devices. Asymmetric performance is caused by the non-uniformity of the carbon nanotube forests on either side of the device, which leads to each side of the cathode performing differently under the same conditions. The likely source of this non-uniformity is the iron catalyst layer that is deposited through a mask during fabrication. CNTs grow from the catalyst layer, and even small differences in catalyst deposition can cause the CNTs to grow differently on each side of the cathode. Improvement to the mask alignment could help improve CNT uniformity and decrease the asymmetric performance issues we observed.

In addition to improvements in performance, a more robust device design is needed to increase device yield. The two most common modes of failure were short circuits due to inconsistent CNT growth and broken panels during device assembly. Panels and latches typically broke during assembly due to the amount of strain placed on the latches when they were raised laterally to hold the panels in place. Increasing the length of the latches would reduce the strain and make devices less prone to failure. Additionally, the panels were placed as close as possible to reduce the voltage requirements, but this made assembly more difficult and prone to failure because the panels and latches were packed closer together. Increasing the panel distance would increase the voltage requirements but could also improve device yield. CNT consistency may be improved with more uniform catalyst deposition, as mentioned above, or by more careful control of chamber conditions in the microwave plasma-enhanced chemical vapor deposition system.

Preliminary radiation exposure experiments showed that CNT field emission was not affected by gamma radiation, even at the high dose rates used. Furthermore, there was no observed damage to the polysilicon device components or the substrate. The dose rate of 45.6 rad(Si)/second is much greater than what a satellite would experience in a low-earth orbit. However, there was a small background current of 7.7 × 10^−9^ A created from ionized molecules in the vacuum chamber. This contribution from this background current is negligible and can be further reduced by testing at higher vacuum conditions, as the radiation experiments described in this paper were conducted at ~1 × 10^−3^ Torr. Furthermore, the effect of background current would be reduced by increasing field emission current and increasing electron transmission to reduce the amount of current that is lost at each grid and increase the current that reaches the anode. With increased electron transmission, transconductance and on/off ratio would also increase, improving device performance.

## Data Availability

Data is contained within the article in the images and plots.
